# Optimal selection of suitable templates in protein interface prediction

**DOI:** 10.1093/bioinformatics/btad510

**Published:** 2023-08-21

**Authors:** Steven Grudman, J Eduardo Fajardo, Andras Fiser

**Affiliations:** Department of Systems and Computational Biology, Albert Einstein College of Medicine, Bronx, NY 10461, USA; Department of Systems and Computational Biology, Albert Einstein College of Medicine, Bronx, NY 10461, USA; Department of Systems and Computational Biology, Albert Einstein College of Medicine, Bronx, NY 10461, USA

## Abstract

**Motivation:**

Molecular-level classification of protein–protein interfaces can greatly assist in functional characterization and rational drug design. The most accurate protein interface predictions rely on finding homologous proteins with known interfaces since most interfaces are conserved within the same protein family. The accuracy of these template-based prediction approaches depends on the correct choice of suitable templates. Choosing the right templates in the immunoglobulin superfamily (IgSF) is challenging because its members share low sequence identity and display a wide range of alternative binding sites despite structural homology.

**Results:**

We present a new approach to predict protein interfaces. First, template-specific, informative evolutionary profiles are established using a mutual information-based approach. Next, based on the similarity of residue level conservation scores derived from the evolutionary profiles, a query protein is hierarchically clustered with all available template proteins in its superfamily with known interface definitions. Once clustered, a subset of the most closely related templates is selected, and an interface prediction is made. These initial interface predictions are subsequently refined by extensive docking. This method was benchmarked on 51 IgSF proteins and can predict nontrivial interfaces of IgSF proteins with an average and median *F*-score of 0.64 and 0.78, respectively. We also provide a way to assess the confidence of the results. The average and median *F*-scores increase to 0.8 and 0.81, respectively, if 27% of low confidence cases and 17% of medium confidence cases are removed. Lastly, we provide residue level interface predictions, protein complexes, and confidence measurements for singletons in the IgSF.

**Availability and implementation:**

Source code is freely available at: https://gitlab.com/fiserlab.org/interdct_with_refinement.

## 1 Introduction

Protein–protein interactions are the primary facilitators of most cellular functions, including but not limited to signal transduction, molecular transport, and immune function (Nelson 2008). Characterizing protein–protein interfaces can assist in rational drug design (Jubb *et al.* 2015), protein classification (Yap *et al.* 2014), and ligand prediction (Yap and Fiser 2016). Experimentally, protein interfaces are derived from X-ray crystallography (Shi 2014) or nuclear magnetic resonance (NMR) spectroscopy (Göbl *et al.* 2014). However, these experimental methods are frequently associated with several challenges such as being labor-intensive, time-consuming, and having limited applicability (Szymczyna *et al.* 2009). Additionally, protein co-crystals are often difficult to obtain for X-ray crystallography, and protein–protein complexes are often prohibitively large for NMR studies (Khafizov *et al.* 2014). As such, computational approaches remain an important alternative to predict protein interfaces. Broadly, the categories used to computationally predict protein interfaces are template-based (Xue *et al.* 2011) and ab initio methods. The latter employs docking methods (Keskin *et al.* 2011) or some combination of docking and feature recognition, often in a machine learning setting (Neuvirth *et al.* 2004). Meanwhile, template-based methods have been shown as the best possible approach when applicable (Martí-Renom *et al.* 2000). However, the availability of suitable homologs is a major bottleneck to this approach. Protein pairs with a sequence identity >35% are considered to be in the “safe zone” and comprise the best possible templates (Rost 1999). Protein pairs with 20%–35% sequence identity are considered to be in the “twilight zone” and have <5% chance of sharing the same structural family (Rost 1999).

To overcome the limitations of any single computational interface prediction method, meta-predictions attempt to combine complementary approaches. For example, the integrated structure-based protein interface prediction (ISPIP) (Walder *et al.* 2022) uses either linear regression, random forest, or XGBoost to combine the predictive strengths of PredUs 2.0, a template-based method (Zhang *et al.* 2011), ISPRED4, a template-free method (Savojardo *et al.* 2017), and DockPred, a docking-based method (Viswanathan *et al.* 2019). ISPIP was trained and evaluated on a diverse set of proteins and was found to outperform all of its input methods, which generated average *F*-scores between 0.38 and 0.41, while ISPIP generated an average *F*-score of 0.52 when using XGBoost (Walder *et al.* 2022). Although this recent method boasts an impressive interface prediction accuracy compared to other alternatives, it is still not accurate enough for practical applications. In interface prediction, one would need to consider how incorrectly an interface can be defined while still capturing its biological function. A recent study addressing this issue reported that for an interface prediction method to be useful, it must capture at least 70% of the true biological interface of the protein (Nandigrami and Fiser 2023). This suggests that the currently available best approaches still do not reach the desired level of accuracy for practical applications.

Protein interfaces are under evolutionary pressure to maintain their function, which is to specifically recognize their cognate ligand. Hence, it is expected that protein interface residues are more conserved than the rest of the protein (Ma *et al.* 2003). However, in practical applications, the conservation signal from interface residues proved to be relatively weak (Caffrey *et al.* 2004). We recently showed that the selection of sequences to include in a multiple sequence alignment (MSA) is a major factor in establishing a strong signal for subsequent conservation analysis. In response to that, we introduced a method, Selection of Alignment by Maximal Mutual Information (SAMMI), that samples hundreds of alternative subsets of related sequences and employs a mutual information-based method to select an MSA with the highest information content (Gil *et al.* 2018, Gil and Fiser 2019). Conservation analysis of these MSAs proved to produce a more characteristic signal than arbitrarily constructed MSAs, for instance, than the default MSAs recovered after a typical PSI-Blast run.

**Figure 1.**
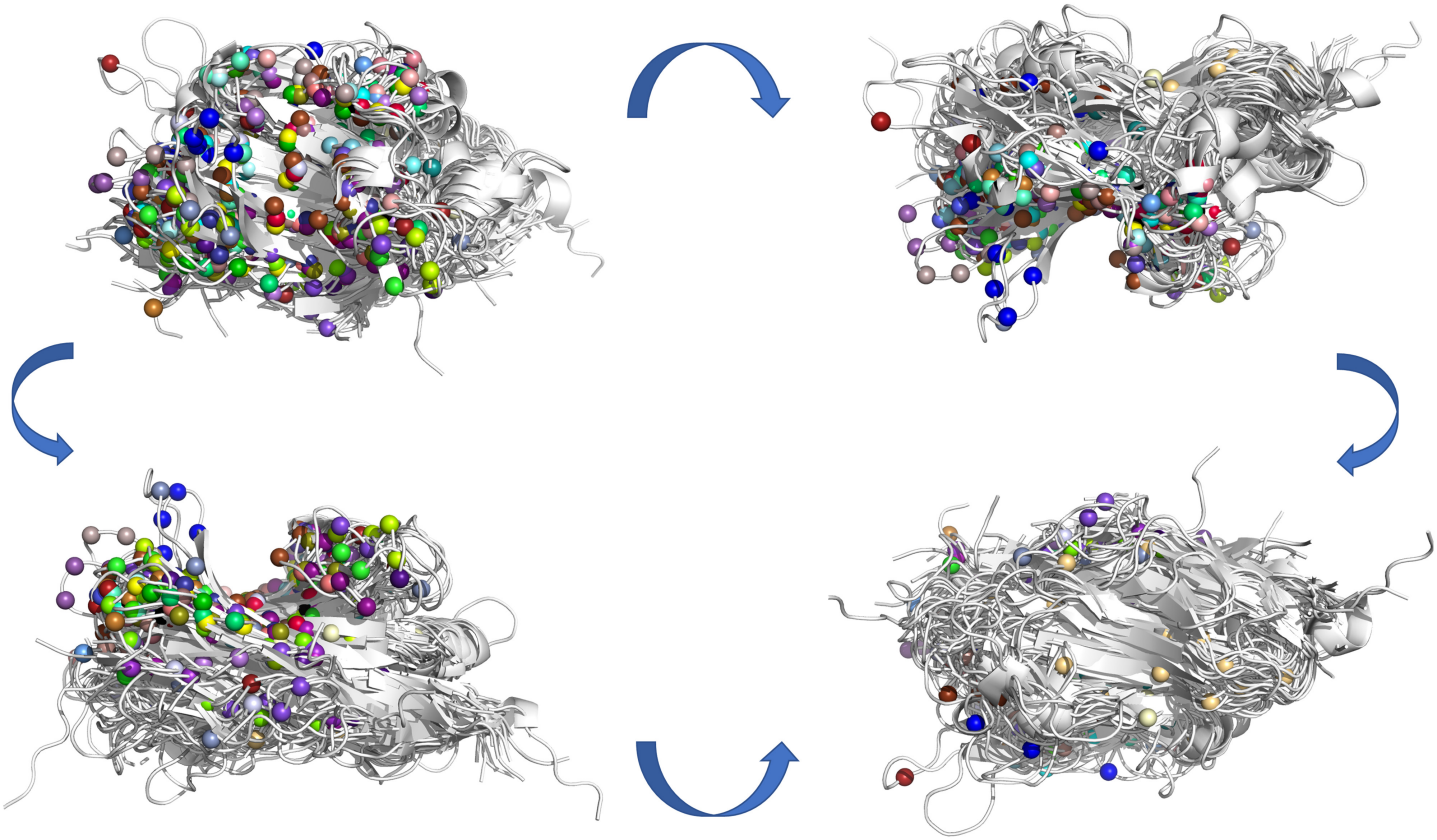
IgSF template interfaces; 90° rotations of all structurally superimposed 51 IgSF protein templates are shown in white. The Cα atoms of each interface residue for all the 51 proteins are represented by spheres. Spheres of the same color belong to the same interface.

In this work, we chose to focus on the IgSF, one of the largest superfamilies in the human genome that specializes in protein recognition. Specifically, we chose to explore “extracellular” IgSF (Yap *et al.* 2014, Gil *et al.* 2020) proteins, which are all IgSF proteins excluding antibodies, major histocompatibility complex proteins, and T-cell receptors. These proteins carry out their functions after establishing interactions with other proteins in *trans* (Barclay 2003) and are of special biomedical interest since they have been implicated in many pathologies including infection, autoimmune disease, and cancer (Karandikar *et al.* 1996, Dermody *et al.* 2009, Dai *et al.* 2014, Tanvetyanon *et al.* 2017). Although some IgSF protein pairs can be clustered in the safe zone of sequence similarity, most share <35% sequence identity even though they all share the same structural fold (Chattopadhyay *et al.* 2009). This super-family also has many crystallized complexes available to derive possible template interface definitions. Meanwhile, the binding interfaces in the IgSF localize diffusely among its members making them hard to predict even when homologs are available (Fig. 1).

Template-based methods are the most reliable approaches for predicting protein interfaces when suitable templates are available and correctly selected (Martí-Renom *et al.* 2000). Identifying structurally related proteins is relatively straightforward nowadays (Fiser 2010); therefore, the single most important factor in these approaches is the correct selection of functionally related template protein(s). Therefore, our approach focuses on identifying the best subset of templates among all known available homologs. In the current work, we hierarchically cluster a query protein with all possible templates using an approach that utilizes interface residue conservation scores, derived from MSAs that were identified by SAMMI. The most characteristic cluster of templates is selected, and an initial binding site prediction is made (Supplementary Fig. S1). Subsequently, docking is employed to produce a physically meaningful final binding site prediction. The protein interface prediction method described in this article can be applied to any group of proteins or families that have known experimental structures available. Proteins that share <35% sequence identity will benefit the most from this method since interfaces for proteins that share >35% sequence identity with at least one template are trivial to predict with high accuracy.

## 2 Materials and methods

### 2.1 Template selection

A list of 51 PDB entries were collected from the Protein Data Bank (Berman 2000). These PDBs were selected if they formed a cognate, *trans*-binding complex and included at least one extracellular IgSF protein (Supplementary Table S1). Although some extracellular IgSF proteins have multiple PDB complexes available, we used only one PDB per protein to not bias the interface prediction method. Noncognate protein complexes, multi binding domain complexes, and complexes facilitated by mutations were excluded.

### 2.2 Interface definition from clustered templates

**Figure 2.**
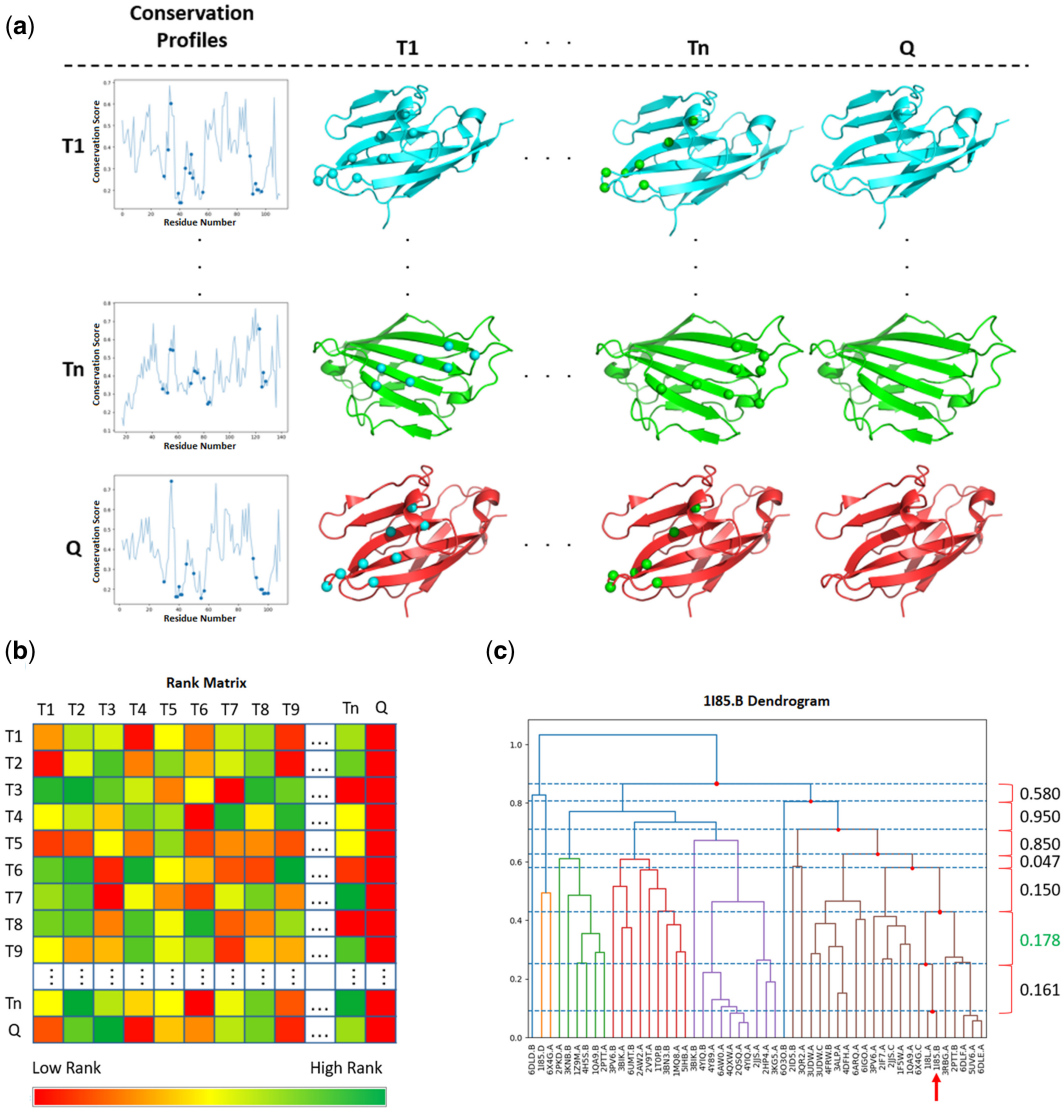
Hierarchical clustering overview. (a) Matrix representation for mapping template interface residues. Template 1 (T1), Template n (Tn), where *n* is the total number of available templates, and the Query (Q) are shown in cyan, green, and red ribbon models, respectively. Interface residues are shown with spheres. Corresponding residue conservation profiles are shown for each protein on the left with blue circles highlighting the interface positions. Template interfaces are shown in the same color as their ribbon diagrams. Interface positions are structurally mapped onto all other templates and the query across each column. (b) Representation of the rank matrix where ranks are calculated from the sum of the top eight most frequently mapped interface conservation scores for all templates and the query across every row. (c) Dendrogram showing the templates and query hierarchically clustered when considering 1I85.B as the query. Dotted blue lines are drawn on all nodes, shown as red dots, containing the query, 1I85.B, with the distances between each node are shown on the right with the largest distance being highlighted in green.

The first step in the algorithm is to assemble a template library, a collection of experimentally determined structures of proteins of the same fold family as the query in complex with a protein ligand. The Interface Contact definition with Adaptable Atom Types (INTERCAAT) program is then applied with default parameters to determine the binding interface residues of each complex (Grudman *et al.* 2022). Next, a MSA is constructed for each template and query proteins. This is accomplished using the SAMMI method, which implements a mutual information based algorithm to optimally select the most informative sequence profile for a given query protein (Gil *et al.* 2018). These MSAs are then used to calculate a conservation score for each residue in a query protein based on the Jensen–Shannon divergence (Capra and Singh 2007). Conservation scores range between 0 and 1 with higher scores representing higher residue conservation. Structural models for query proteins are gathered from AlphaFold (Jumper *et al.* 2021) when no crystal or NMR structures are available, while structures for template proteins are all collected from the Protein Data Bank (Berman 2000). In parallel, the binding site information obtained from each template protein in the INTERCAAT step is mapped onto all other proteins in a pairwise manner by performing a structural superposition using the *align3d* function from MODELLER 10.2 (Fiser and Šali 2003) assigning the annotation to structurally equivalent residues. This step effectively represents single-template interface predictions. Any mapped residues that have a solvent accessible surface area (SASA) <5 Ǻ2, calculated by NACCESS using default probe size (Hubbard and Thornton 1993), are assumed to be buried and discarded. These single template interface predictions can be used to construct a matrix M where each entry *i*, *j* represents the predicted interface residues of protein *j* based on the annotated interface residues of protein *i* (Fig. 2a). To quantify this matrix, the interface predictions are converted into numerical entries by summing the predicted interface residue conservation scores as calculated from the corresponding MSAs established by the SAMMI method. Only the top eight most frequently mapped residues (Supplementary Fig. S2) are summed up because the smallest interface size identified by INTERCAAT (Grudman *et al.* 2022) among all known templates is eight residues long. The values on each row of this matrix are then ranked from highest to lowest according to the sum of the conservation scores (Fig. 2b) resulting in a rank matrix. Next, a dissimilarity matrix is calculated based on the correlation between pairs of rows of the rank matrix. This distance matrix is then used to perform hierarchical clustering and produce a dendrogram that includes all the template proteins plus the query. Both, generation of the distance matrix and the clustering process can be accomplished in a single step using the scipy.cluster.hierarchy.linkage python function using the “weighted” method and “correlation” metric (Virtanen *et al.* 2020). For easy visualization, the result can be displayed as a dendrogram using the scipy.cluster.hierarchy.dendrogram python function (Virtanen *et al.* 2020) (Fig. 2c). Next, a subset of templates that are best clustered with the query must be selected. To determine which subset to take, a list of all the internal nodes that subsume a subtree that includes the query is prepared and the distances between all pairs of contiguous nodes in the list containing the query are calculated. The pair of nodes with the largest distance is identified and the templates in the subtree rooted by the lower node are used to predict the queries interface (Fig. 2c). Each selected template is aligned to the query using the *align3d* function from MODELLER 10.2 (Fiser and Šali 2003) and all template interface residues are mapped onto the query. Mapped residues that are shared by at least 20% of the selected templates (Supplementary Fig. S3) and have a SASA ≥5 Ǻ^2^, calculated by NACCESS using default probe size (Hubbard and Thornton 1993), compose the initial interface definition: *inter*face *d*efinition from *c*lustered *t*emplates or interDCT. It is trivial to predict a protein interface if it shares more than 35% sequence identity with any template (Rost 1999). For these proteins, interface definitions can be predicted by mapping the residues of the highest sequence identity template, along with any other templates sharing >35% sequence identity with the query and within 20% sequence identity of the best template, onto the query. All mapped interface residues must have SASA ≥5 Ǻ^2^ calculated by NACCESS using default probe size (Hubbard and Thornton 1993).

### 2.3 Refinement

**Figure 3.**
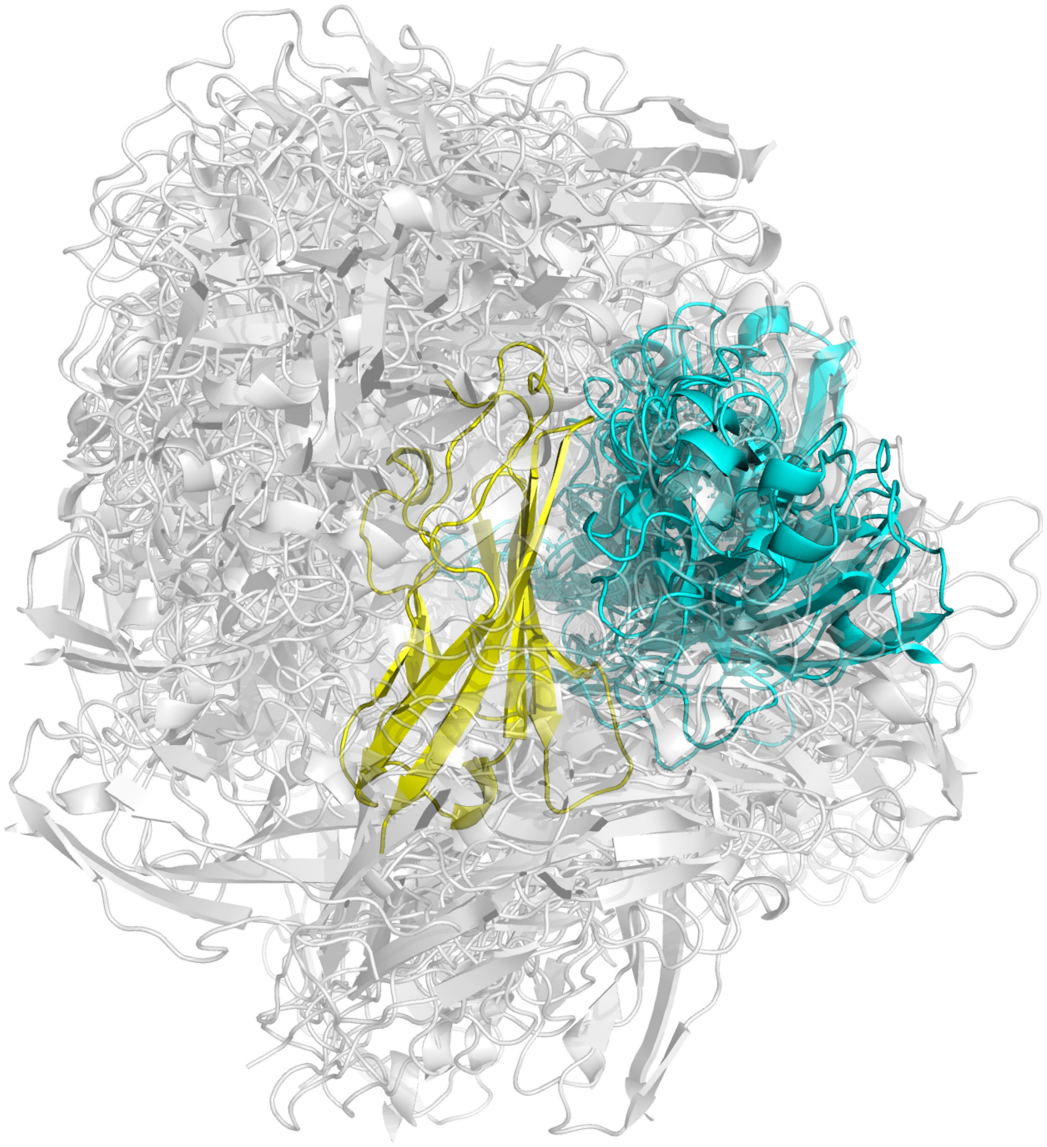
Illustration of the refinement step. The query protein in yellow is shown forming 200 complexes with one of the ligand probes shown in grey or blue. The docked probes in blue have the highest *F*-scores with reference to our interDCT. These complexes are used to refine the initial interDCT definition.

InterDCT predictions are generally slightly larger (average interface size = 17.9 residues) than cognate IgSF interfaces (average interface size = 15.8 residues). To home in on a more accurate interface prediction that precisely accommodates a single ligand, we proceed to dock our query with 13 different, unrelated protein probes (Fig. 3). Six of these probes have Ig folds while the other seven have distinct, randomly selected, small protein folds. The probes we selected are the same ones that were recently used to confirm that proteins typically have binding supersites (Viswanathan *et al.* 2019). The ZDOCK docking program (Keskin *et al.* 2011) by default creates 2000 docked complexes per probe, so for 13 probes, ZDOCK will create 26 000 docked complexes. For each of the 26 000 complexes, we calculated the interface definitions by INTERCAAT (Grudman *et al.* 2022). This allows us to identify the top 100 complexes with the largest residue overlap with our initial interDCT definition (Supplementary Fig. S4). These complexes are used to calculate interface residue “true positive” and “false positive” frequencies which are defined as residues found and not found in the interDCT, respectively. Residues that have a true positive frequency >50% and a false positive frequency >35% (Supplementary Fig. S5) constitute the final refined interface definition. We explored if we could decrease the number of calculations needed for this refinement step by checking if we could reduce the number of probes or the number of generated ZDOCK complexes without decreasing the accuracy of the method. But we found that it is still best to use all 13 probes and all 2000 complexes (Supplementary Fig. S6). After refinement, the average interface size decreases slightly from 17.9 to 17.1 residues. The reason the interface size does not decrease more is due to the nature of the *F*-score which punishes missing true positive residues more than having extra false positive residues.

### 2.4 Protein Interface definition

The program INTERCAAT was used with default settings to determine protein interfaces (Grudman et al. 2022). INTERCAAT employs Voronoi tessellation to identify atoms that share a hyperplane and whose distance is less than the sum of each atom’s Van der Waals radii plus the diameter of a solvent molecule. Interacting atoms are then classified and interactions are filtered based on compatibility.

### 2.5 F-score calculation

*F*-scores were calculated using the standard *F*_1_-score formula: $\frac{2*\mathrm{tp}}{2*\mathrm{tp}+\mathrm{fp}+\mathrm{fn}}$ where tp = true positives, fp = false positives, and fn = false negatives.

## 3 Results

### 3.1 Benchmarking

**Figure 4.**
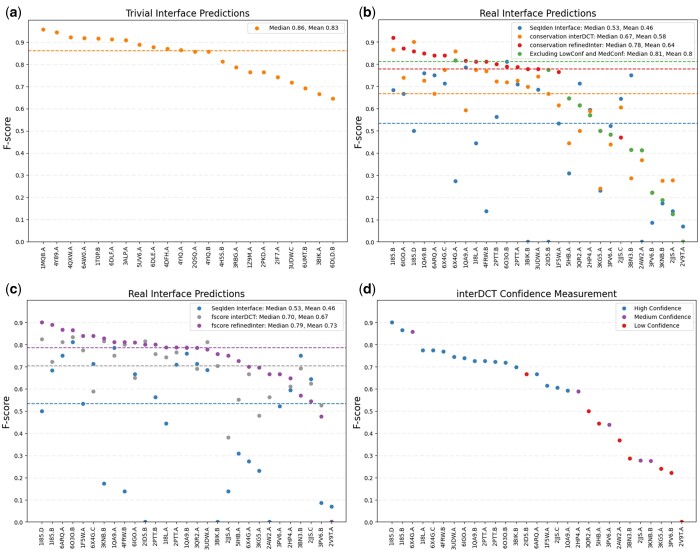
Results of predicting each of the 51 known IgSF protein interfaces using a leave-one-out approach. PDB codes of the tested proteins are on the *X*-axis and statistical *F*-scores of performances are on the *Y*-axis. Dotted lines are plotted at the medians of each *F*-score distribution. (a) *F*-scores of the prediction results of the trivial protein interfaces. (b) *F*-scores of the prediction results of nontrivial protein interfaces. Seqiden results, shown in blue, are calculated as in panel (a) but for the nontrivial cases. The conservation interDCT results, shown in orange, are the initial interface predictions *F*-scores obtained before refinement. The conservation refinedInter (Refined interface), shown in red, are the conservation interDCT *F*-scores after refinement. The green distribution is overlaid on top of the any of the red data points that have low or medium confidence. (c) Like in (b), Seqiden results shown in blue are calculated as in panel (a) but for the nontrivial cases. The *F*-score interDCT, shown in grey, uses the interface definitions of the nontrivial proteins instead conservation scores to optimally cluster all templates and predict the theoretical best interDCTs. The *F*-score refinedInter, shown in purple, are the *F*-score interDCT *F*-scores after refinement. (d) Confidence measurements of the 29 interDCT predictions for the nontrivial proteins.

To evaluate the accuracy of our interface prediction method, we performed a leave-one-out cross validation approach using our set of 51 IgSF protein complexes (Supplementary Table S1). A leave-one-out approach pretends to not know one of our templates interfaces, which we call the query, and uses the other 50 templates to predict its interface. *F*-scores were used to evaluate interface predictions by comparing our predictions with the templates actual interface definition according to INTERCAAT (Grudman *et al.* 2022). Out of the 51 IgSF complexes, 22 and 29 turned out to have interfaces that were trivial and nontrivial to predict respectively, i.e. the trivial cases shared more than 35% sequence identity with at least one template (Fig. 4a), while the nontrivial cases shared <35% sequence identity with any of the templates (Fig. 4b).

To establish a baseline for our approach, we calculated naïve interface predictions for each nontrivial protein using a single template with the highest sequence identity to the query. The average and median *F*-scores of the naïve approach were 0.46 and 0.53, respectively. To establish the theoretical upper limits of our method, we hierarchically clustered our templates using a distance matrix prepared as before but using the *F*-scores of pairwise binding site similarities directly instead of conservation scores. We then used the resulting dendrogram to make the hypothetical best possible interDCT predictions (Fig. 4c). After refinement, the medians of the *F*-scores for the hypothetical best interDCTs (0.79) (Fig. 4c) and the *F*-scores derived from the real conservation interDCTs (0.78) (Fig. 4b) perform comparably well. However, when we compare overall mean values, the hypothetical best method outperforms our method (*F*-scores of 0.73 versus 0.64) since our method is unable to optimally cluster and select the best templates for about 1/3 of the nontrivial proteins. To address some of the failures in our method, we sought to distinguish interface predictions by establishing a confidence measure.

### 3.2 Confidence measurement

Due to the limited number of templates available and the heterogeneity of the IgSF superfamily, it is possible that no suitable template(s) currently exists that can accurately predict the interfaces for certain queries. It is also possible that our method may sometimes fail to cluster a query to the correct subset of templates. As such, we created a confidence measurement that can assign interDCT predictions with either a high, medium, or low confidence. The concept behind the confidence measure is that templates that are clustered with the query are expected to be informative about each other as well. To gauge this, we can measure how consistently the templates predict each other’s interface. A high success rate of predicting interfaces among the templates would suggest that these selected templates are more consistent and more likely that they will deliver a good prediction to the query as well. Besides overall average success rate we also monitor the standard deviation of these predictions to account for outliers among templates.

First, we cluster a new set of templates to our query by going through the interDCT method, but instead of selecting the top eight most frequently mapped residues, we choose eight random interface residues instead (Supplementary Fig. S2). We define this as selecting alternative interDCT templates. Again, we always choose eight residues because the smallest interface size identified by INTERCAAT (Grudman *et al.* 2022) among all known templates is eight residues long. Next, we systematically predict each of the clustered templates’ own interfaces using a leave one out approach using only the group of clustered templates. Since we know each template’s true interface, we can compare it with the interface’s predicted using the leave one out method and quantify the comparison as an *F*-score. We then average and take the standard deviations of all interface prediction *F*-scores over all clustered templates. This is repeated 10 times selecting 10 alternatively clustered interDCT templates. The resulting average of *F*-scores and average of standard deviations are separately averaged, which we will define as template reliability and template inconsistency, respectively. If template reliability >0.525 and template inconsistency <0.175, the interDCT prediction is deemed to have high confidence. If template reliability is either <0.525 or template inconsistency >0.175, the interDCT prediction is deemed to have medium confidence. If template reliability <0.525 and template inconsistency >0.175, the interDCT prediction is deemed to have low confidence (Fig. 4d). These cutoffs were calibrated by checking the confidences of each query after incrementing template reliability and template inconsistency by intervals of 0.025.

Note that these distinctions between high, medium, and low confidence were optimized for this set of templates. If another set of templates is used, these parameters will likely need to be adjusted. The results of implementing the confidence metric improves the average and median *F*-score of 0.64 and 0.78 to 0.8 and 0.81, respectively, if all medium confidence (17%) and all low confidence (28%) cases are removed (Fig. 4b).

## 4 Discussion

In this article, we explored how much one can improve template-based interface prediction for proteins whose homologs share low sequence identity. The method developed in this article was motivated by the recent observation that the minimum interface prediction accuracy needs to be at least 70% correct to capture the biological function of a protein, as assessed by its ability to reliably recognize its cognate binding partner(s) (Nandigrami and Fiser 2023). This goal is a trivial task for proteins that share more than 35% sequence identity with a functionally characterized known template (Fig. 4a). However, for proteins that share <35% sequence identity with known templates, this question quickly becomes challenging. The core concept of our approach is to identify the best subset of functionally related protein templates available for a query protein by analyzing the similarities of their binding site conservation profiles. Then, the interface residues from the identified templates are mapped onto the query, and the initial prediction is refined through docking. The method discussed here can predict the interfaces of nontrivial IgSF proteins with a mean *F*-score of 0.64 and a median *F*-score of 0.78 (Fig. 4b). After excluding all low and medium confidence predictions, the mean *F*-score increased to 0.8 and the median *F*-score increased to 0.81 (Fig. 4b).

In addition to outperforming meta methods for interface prediction, such as the recently published ISPIP method (Walder *et al.* 2022), our method was also tested against another state-of-the-art method, molecular surface interaction fingerprinting (MaSIF), specifically the MaSIF-site application which is the protein–protein interaction application (Gainza *et al.* 2020). MaSIF applies a deep learning algorithm to capture geometric and chemical features to predict ligand binding sites regardless of a protein sequence or structural fold. It was tested against other top-performing interface prediction methods such as SPPIDER (Porollo and Meller 2007) and PSIVER (Murakami and Mizuguchi 2010), and it outperformed them. Our method achieved a median ROC AUC per protein of 0.81 whereas SPPIDER and PSIVER achieved median ROC AUCs per protein of 0.65 and 0.62, respectively. We tested MaSIF’s ability to predict the interfaces of our template database excluding the proteins used in MaSIF’s training set (1I8L.A, 1QA9.A, 1QA9.B, 1T0P.B, 2AW2.A, 2JJS.A, 2PPT.A, 2PTT.B, 3ALP.A, 3BN3.B, 3PV6.B, 4H5S.B). However, MaSIF does not explicitly assign interface residues like we do; instead, MaSIF computes a discretized mesh around a protein structure with a water probe radius of 1.5 Å and vertexes being separated by 1.0 Ǻ. Each vertex is assigned an interface score between 0 and 1 with scores closer to 1 having a higher interface propensity (Gainza *et al.* 2020). To make MaSIF-site comparable with our method, the MaSIF’s interface representation was converted into residue level interface assignments. This was accomplished by assigning each vertex to a residue if its distance to that residue was below 2.8 Ǻ, the diameter of a solvent molecule. We then tested a range of interface score cutoffs (ifaceCutoff) between 0.6 and 0.85 and the minimum number of vertexes needed to be assigned to a residue for it to be considered as part of the interface (minVertexCutoff) between 0 and 18. We then compared the results with our naïve approach of predicting an interface, namely mapping the interface of the highest sequence identity template onto the query. We found that even this naïve approach significantly outperformed MaSIF for both trivial and nontrivial IgSF proteins (Supplementary Fig. S7). MaSIF is an advanced *ab initio* interface prediction method; however, these results reinforce the fact that template-based interface prediction methods are far superior when it is possible to use them.

Contrary to original expectations, using only the conservation scores of interface residues did not predict the best available templates. This is because the average conservation scores were not significantly different between interface and noninterface residues, in agreement with what was observed before by others (Caffrey *et al.* 2004). However, we noticed that a meaningful relationship can be established between the interface conservation profiles. These relationships in conservation levels were captured by mapping all possible template interface residues to all other templates and the query. The corresponding conservations scores were summed and ranked resulting in a rank matrix (Fig. 2b). Mapping the interface residues of protein *i* onto protein *j* is not equivalent to mapping the interface residues of protein *j* onto protein *i*. For this reason, the rank matrix is not symmetric. By comparing the rows of the matrix, some proteins can immediately be seen as being more related than others. For example, template 1, template 2, and the query can easily be recognized as being related due to the rank similarities between corresponding rows (Fig. 2b). After establishing which templates are most related to a query, an interDCT prediction can be made by collecting the eight most frequently mapped residues among these templates. Collecting the most frequently mapped residues was found to provide a better signal than choosing eight randomly mapped residues (Supplementary Fig. S8). This is because these residues are the most conserved interface residues among all template proteins and therefore carry the most information. We consider any interDCT predictions derived from mapping random interface residues as alternative interDCT predictions. We use the templates clustered from these alternative interDCTs for our confidence measurement.

Another question of possible interest is how we can cluster a query protein without knowing its interface. This again can be answered by looking at the rank matrix (Fig. 2b). One can notice that the last column of the rank matrix is all red representing the lowest rank. This is by no means a coincidence. Since we do not know the interface of the query, it cannot be mapped onto any of the template proteins. As such, the sum of predicted interface residue conservation scores will always be zero. This becomes important when we convert the rank matrix into dissimilarity matrix, which is used to hierarchically cluster the query and templates. The conversion is done by calculating all pairwise distances between all rows in the rank matrix. Since the last term in each row is always the same, it will cancel out in the distance calculation. Indeed, knowing the interface of the query does not even improve this method. The interDCT predictions are essentially indistinguishable irrespective of whether the query proteins interface is known or unknown (Supplementary Fig. S9).

As in the case of other template-based methods, the effectiveness of our interface prediction method depends upon the quantity of relevant templates available. Additionally, this method relies on the quality of models derived from AlphaFold (Jumper *et al.* 2021), when no experimentally derived structural templates are available, and the quality of conservation scores derived from SAMMI (Gil *et al.* 2018). Unreliable predictions from either of these input methods would severely impact the prediction accuracies. However, to use the method for a query with no experimentally derived structure, one would have to rely on a structural model, which may decrease the overall reliability of the method. Another critical component of the approach is the quality of the conservation scores, which were shown to heavily depend on the composition of the MSAs being used. This is why we chose to use SAMMI since it has proven itself to be one of the most reliable methods for selecting MSAs with informative sequence compositions (Gil *et al.* 2018).

The template-based binding site prediction method discussed in this article can be perceived as a constrained docking approach. Using prior knowledge to guide docking is not a new idea. The High Ambiguity Driven protein–protein Docking method (HADDOCK) pioneered the concept by utilizing NMR-derived restraints to concentrate docking around relevant regions of a protein, effectively reducing the docking sampling space (Dominguez *et al.* 2003). Given this, we sought to use our interDCT results to guide the selection of the best complexes generated by ZDOCK and distill a final interface prediction from those selected complexes by residue frequency.

As a practical result, we provide interface predictions, along with confidence measures, for 78 of the extracellular IgSF singletons identified by the Protein Interactions Calibrated hidden Markov model Tree (PICTree) method (Yap *et al.* 2014) (Supplementary Table S2). Singletons in the IgSF are the most difficult cases when trying to determine suitable templates (Supplementary Table S2). These proteins are generally understudied even though many of them are biomedically important (Edwards *et al.* 2011). For the 78 IgSF singletons, we predicted their N terminal domain interfaces with high, medium, and low confidence in 45, 16, and 17 of the cases, respectively. ZDOCK complexes that best reflect our final interface prediction for the singletons are provided (https://gitlab.com/fiserlab.org/interdct_with_refinement in the bestComplexes directory).

## Supplementary Material

btad510_Supplementary_Data

## Data Availability

Source code is freely available at: https://gitlab.com/fiserlab.org/interdct_with_refinement.
