# Supplementary material for: Optimal selection of suitable templates in protein interface prediction
Source: Bioinformatics. 2023 Aug 21;39(9):btad510. doi: 10.1093/bioinformatics/btad510 (PMC10491951; doi:10.1093/bioinformatics/btad510)
Supplement: btad510_Supplementary_Data [file btad510_supplementary_data.zip › Supplemental Figures-6-22-2023.docx]

**Supplemental Figures:**

**
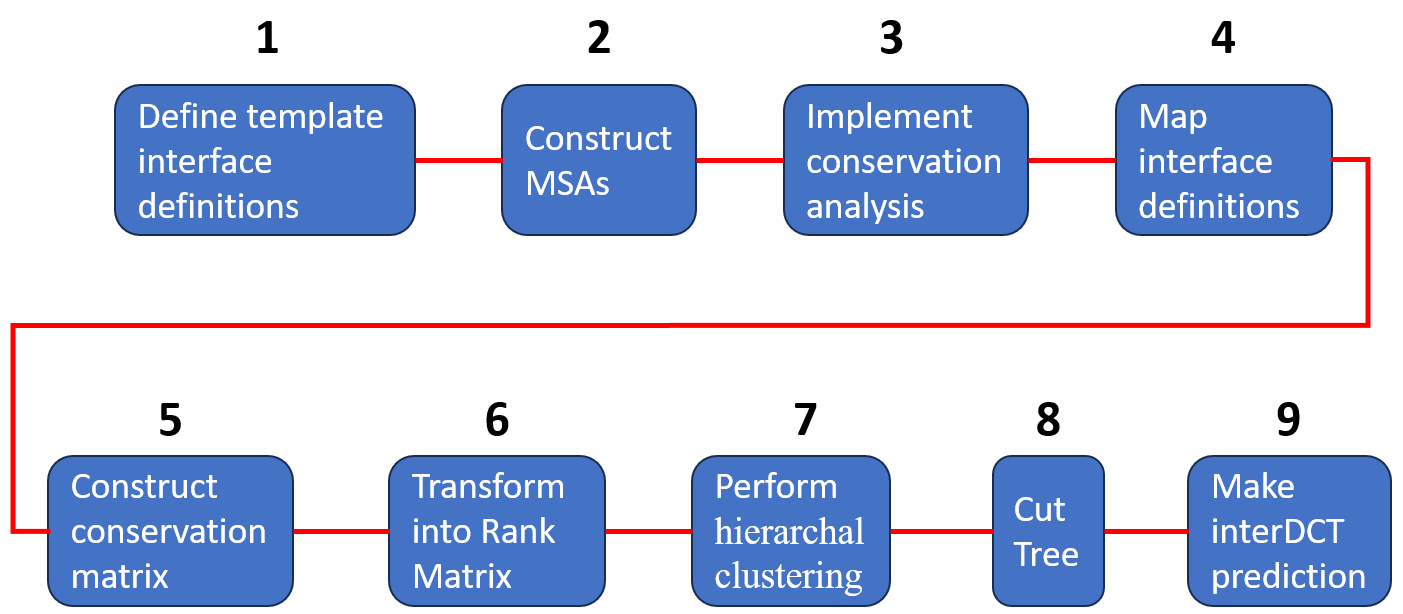
**

**Supplemental Figure 1:** Flowchart of interDCT method. **1)** Use INTERCAAT to define template interfaces. **2)** Construct MSAs for all templates and the query using SAMMI. **3)** Implement conservation analysis on all MSAs using Jensen Shannon Divergence. **4)** Make pairwise structural alignments of every template and the query with one another using MODELLER and map interface residues onto structurally equivalent residues effectively making single template interface predictions. **5)** Convert these interface predictions into a conservation matrix using the residue conservations scores found in step 3. **6)** Transform the conservation matrix into a rank matrix by ranking all conservation scores along every row of the matrix from highest to lowest. **7)** Perform hierarchal clustering using the scipy.cluster.hierarchy.linkage python function with the rank matrix as the input. **8)** Calculate the distance between pairs of contiguous nodes containing the query. Find the pair of nodes with the largest distance and cut the tree at the lower node. **9)** Map the interface residues of the selected templates onto the query making an interDCT prediction.


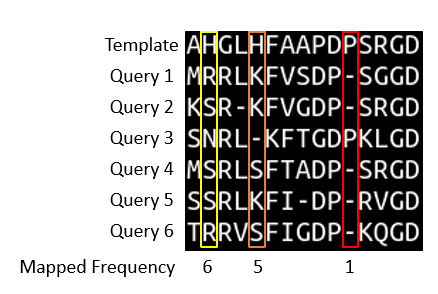


**Supplemental Figure 2:** Illustration of mapping residue frequencies. Interface residues of the template (shown in yellow, orange, and red) are mapped to queries 1-6. Gaps may appear if no equivalent residue exists after the structure-based alignment, which would decrease the mapped frequencies. Additionally, interface residues will not map if the corresponding residue is not exposed (SASA >= 5 Ǻ^2^).


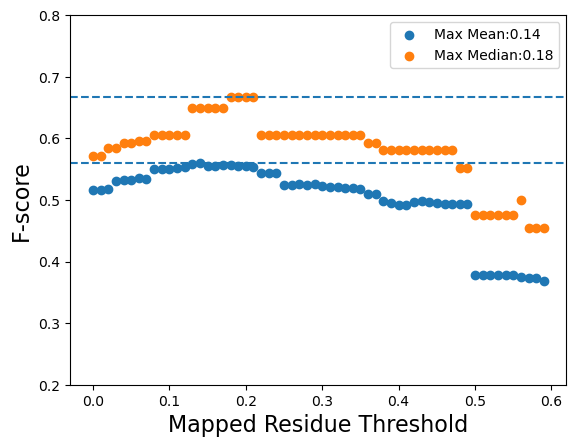


**Supplemental Figure 3:** The group of selected templates are used to make the interDCT prediction. To make the prediction, template residues are mapped onto the query structure. Template residues are accepted if they map to the same query residue a certain percentage of times. To find this optimal limit, we mapped residue frequencies from 0 to 60% for all templates using the leave one out approach. The average results are plotted above.


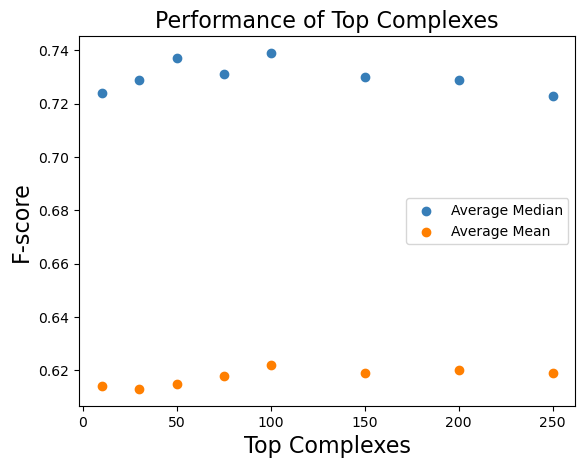


**Supplemental Figure 4:** Optimization of refinement step for how many top models to pick. Various numbers of top models were selected and tested on all templates using a leave one out approach.


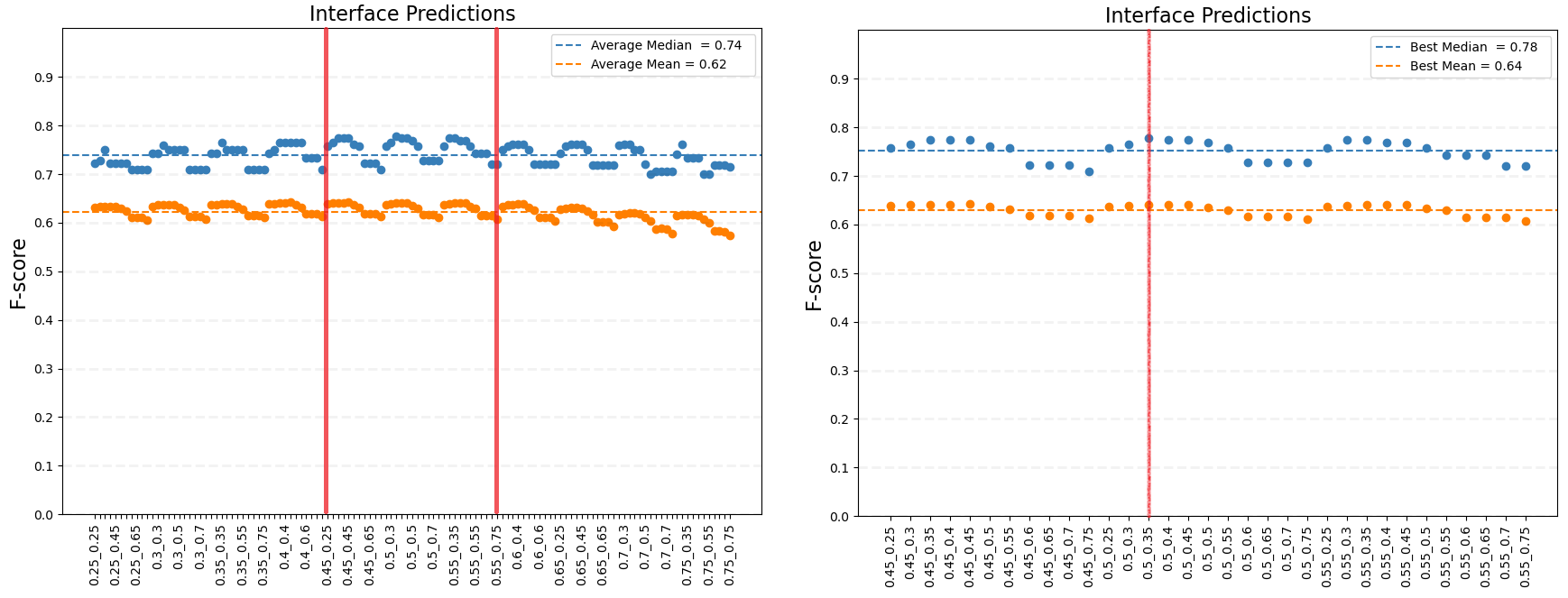


**Supplemental Figure 5:** Optimization of refinement step to find the optimal values for true positive and false positive frequencies when selecting docked poses. On the left, all true positive and false positive frequencies from 25% to 75% were tested in intervals of 5%. The section between the red lines on the left is zoomed in on the right to show the best true positive and false positive frequencies of 50% and 35% respectively shown with the dotted red line.


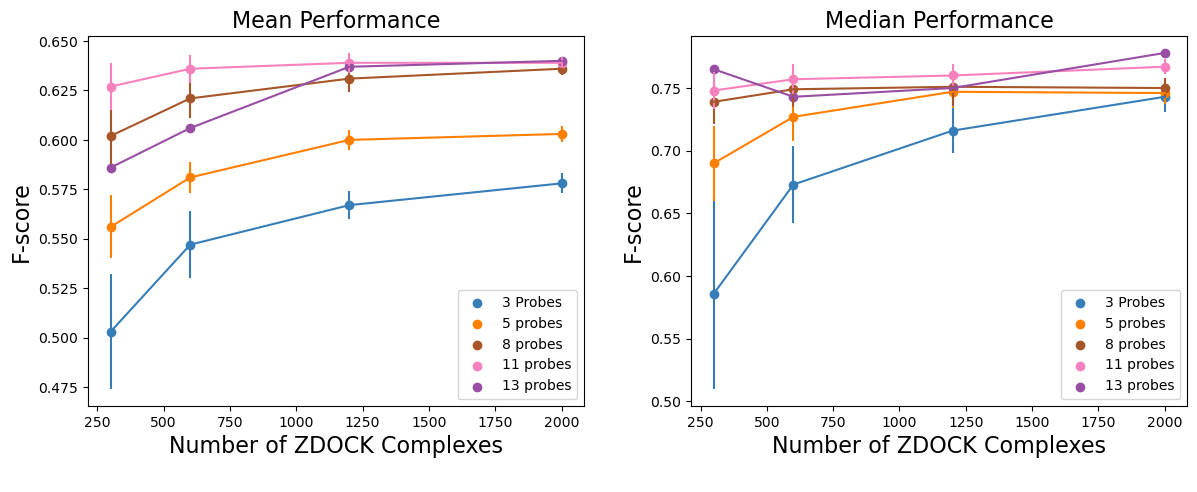


**Supplemental Figure 6:** Optimization of refinement step to determine how many probes should be docked and how many ZDOCK complexes should be generated. When using less than 13 probes all possible probe combinations were enumerated. For example, when testing the performance of 8 probes we calculated the mean and median fscores for all 13C8 combinations. Notice that as the number of ZDOCK complexes increases the standard deviation decreases regardless of how many probes are used.


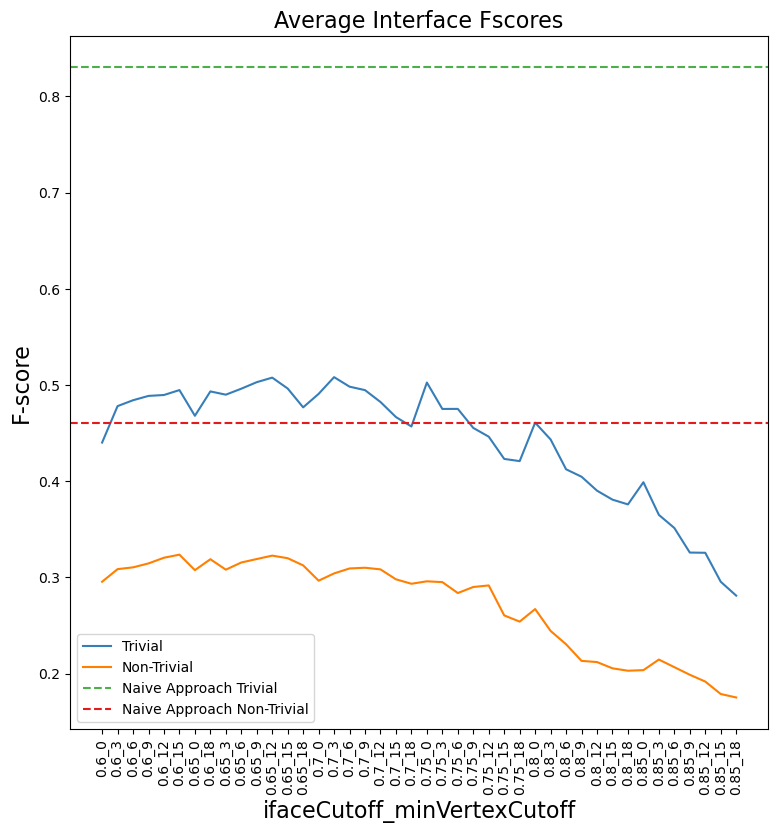


**Supplemental Figure 7:** Ability of MaSIF to predict the interfaces of the trivial and non-trivial IgSF proteins in our template database excluding those used to train MsSIF. Several parameters along the x axis were tested to try and optimize MaSIFs ability to assign protein interface residues; However, regardless of the parameters, MaSIF significantly underperformed compared to the naïve approach to predicting trivial and nontrivial protein interfaces.

**
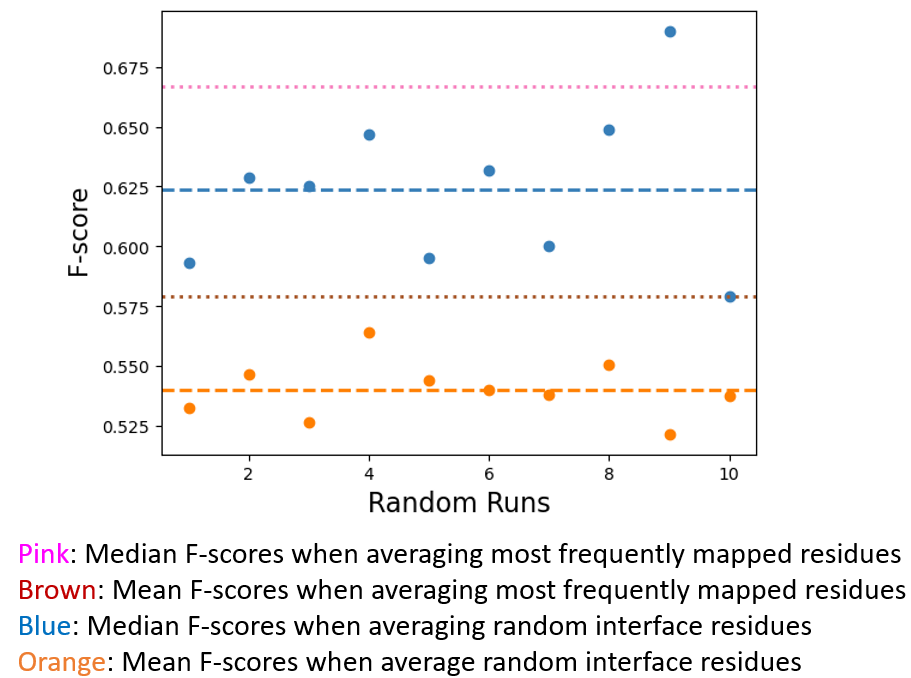
**

**Supplemental Figure 8:** Comparison of inderDCT prediction accuracy using either the most frequently mapped residues or random mapped residues. 10 independent runs were done to predict 10 different alternative interDCTs shown by the blue and orange dots. Only one possible interDCT prediction exists when using the most frequently mapped residues which is shown by the pink and brown dashed lines.


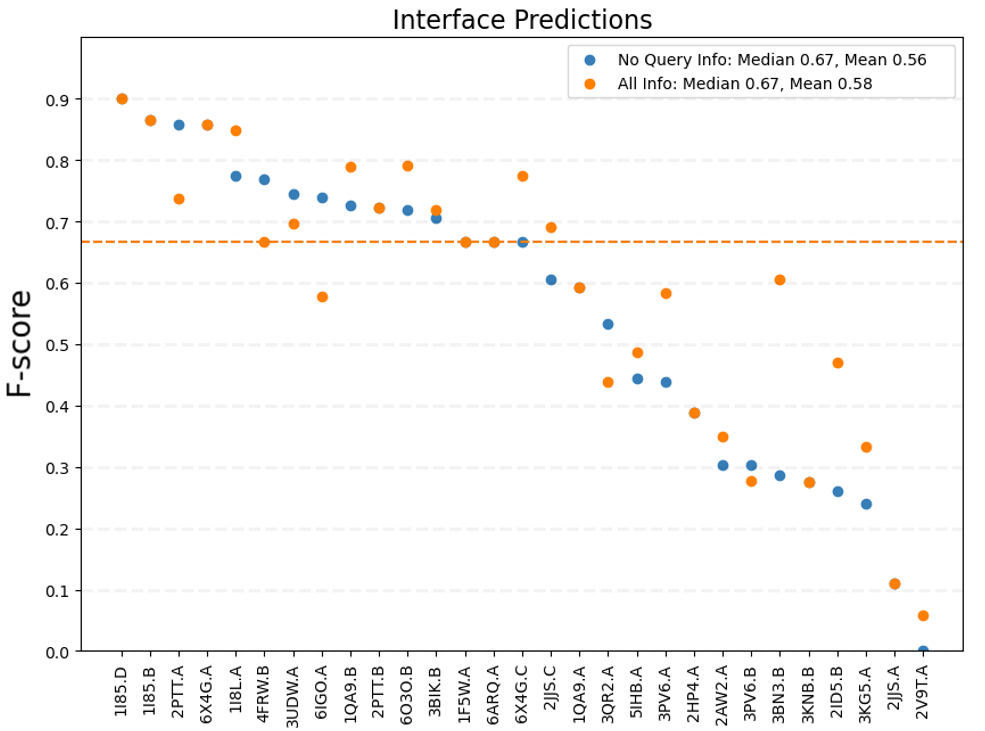


**Supplemental Figure 9:** InterDCT predictions when the queries interface is unknown, shown in blue, and when the queries interface is known, shown in orange.
